# GLUT1 and lactose synthetase are critical genes for lactose synthesis in lactating sows

**DOI:** 10.1186/s12986-018-0276-9

**Published:** 2018-06-13

**Authors:** Yinzhi Zhang, Shihai Zhang, Wutai Guan, Fang Chen, Lin Cheng, Yantao Lv, Jun Chen

**Affiliations:** 1 Guangdong Province Key Laboratory of Animal Nutrition Control, College of Animal Science, South China Agricultural University, Guangzhou, China; 2 College of Animal Science and National Engineering Research Center for Breeding Swine Industry, South China Agricultural University, Wushan Avenue, Tianhe District, Guangzhou, 510642 China

**Keywords:** Lactating sows, Lactose synthesis, Milk, Glucose transportation, Lactose synthetase

## Abstract

**Background:**

Lactose synthesis rate is an important factor in milk production and quality in mammals. Understanding the lactose synthesis mechanism is crucial for the improvement of milk quantity and quality. However, research on the temporal gene changes regarding lactose synthesis during the whole lactation is still limited. The objective of this study was to determine gene expression profiles related to lactose synthesis in sows during lactation, and further identify the critical steps or key factors in the lactose synthesis pathway.

**Methods:**

To determine the temporal change of factors related to lactose synthesis in sows, milk from eight multiparous Yorkshire sows (parity 3 to 6) was collected at 0 h, 2 h, 6 h, 12 h, 24 h, day 2, 3, 4, 7, 14, and 21 after birth of the first piglet. Lactose content, prolactin and progesterone concentration, and gene or protein expression related to lactose synthesis were measured.

**Results:**

The lactose yield increased gradually from D2 to D21 and reached a maximum at D14 (3-fold from D2) during lactation (*P* < 0.05). A similar trend was observed in IGF-1 and insulin concentrations in milk, both of which were greatest at D3 with a subsequent decrease during middle to late lactation. Conversely, milk prolactin and progesterone concentrations moderately decreased with the progression of lactation. The mRNA or protein expressions related to glucose transportation (GLUT1), glucose-galactose interconversion (HK1 and UGP2), UDP-galactose transportation (SLC35A2), and lactose synthetase (LALBA and B4GALT1) in the lactose synthesis pathway were significantly upregulated during early to middle lactation and plateaued by late lactation (*P* < 0.05).

**Conclusions:**

These novel findings suggest that the increased lactose synthesis in lactation was related to the coordinated upregulation of genes or enzymes in the lactose synthesis pathway, and glucose transportation (GLUT1) and lactose synthetase (LALBA and B4GALT1) might be the critical steps in the lactose synthesis pathway of sows during lactation.

## Background

Milk yield and composition of sows is crucial for growth and performance of their piglets [1]. In past decades, genetic selection and improved management have increased the litter size and growth rate of piglets, resulting in an increased nutrient demand by neonates from nursing sows [2]. Restriction of the quality and/or quantity of sow milk could limit the growth potential of suckling piglets, and improvements in milk yield and quality in sows is necessary to prevent such limitations [3–6].

Sow milk contains approximately 5.5% lactose, 4.5% protein, and 6% fat [7–9]. As the primary osmotic agent in milk, lactose, rather than protein or fat, is the major factor influencing milk volume [10] by pulling water into the Golgi vesicles [11, 12]. Furthermore, lactose yield is not only highly correlated with milk yield, but also influences milk fat and protein yield [13, 14]. Thus, understanding the lactose synthesis mechanism is crucial for the improvement of milk quantity and quality.

Lactose synthesis is a complicated process that requires the coordination of many genes encoding for enzymes involved in glucose uptake [15–18], glucose-galactose interconversion [11, 19], UDP-galactose transportation [11, 20], and synthesis of lactose [12, 21–24]. Glucose is transported into mammary epithelial cells from blood by glucose transporters and phosphorylated into glucose-6-phosphate. The glucose-6-phosphate is then converted into UDP-galactose by phosphoglucomutases (PGMs), UDP-glucose pyrophosphorylase 2 (UGP2), galactose-1-phosphate uridylyltransferase (GALT), and UDP-galactose-4-epimerase (GALE). Ultimately, UDP-galactose and glucose in the cytoplasm are transported into Golgi bodies through glucose transporters and UDP-galactose transporters, where lactose is synthesized through lactose synthase composed of β1,4-galactosyltransferase1(B4GALT1) and α-lactalbumin (LALBA). Almost all of the glucose utilized by lactose synthesis in the mammary gland is transported from the blood by glucose transporters [25–27], and glucose transportation into mammary epithelial cells may be the rate-limiting steps for lactose synthesis [28, 29]. This has been demonstrated in rat [30], and goat [31] models during lactation. A previous study in our lab also demonstrated that GLUT1 was the dominant glucose transporter in sow mammary gland from late pregnancy to peak lactation [32, 33]. However, Xiao and Cant reported that hexokinase may be the rate-limiting step in the lactose synthesis pathway [34]. In human models, the gene regulating UDP-galactose conversion and transportation may control the potential critical process in initiation of lactose synthesis [11]. Previous reports have mainly focused on the transitional change of genes between pregnancy and lactation [32, 33, 35–39]. However, research on the temporal gene changes regarding lactose synthesis during the whole lactation is still limited, especially in lactating sows.

Therefore, the objective of this study was to determine gene expression profiles related to lactose synthesis in sows during lactation, and further identify the critical genes or key factors in the lactose synthesis pathway.

## Methods

### Animals and milk collection

A total of 8 multiparous sows (Yorkshire, 3 to 6 parities) were selected from the Changjiang Swine Breeding Center in Guangdong Province (China) and were managed by standard procedures. The diets were corn-soybean meal based and were formulated to meet or exceed nutrient requirements for lactating sows recommended by the National Research Council (NRC) [40]. The diet compositions and nutrient contents are presented in Table 1.

**Table 1:** Composition and nutrient content in diet

Ingredients	Content, g/kg	Nutrients, unit	Calculated value
Corn	470.6	DE, MJ/kg	14.43
Wheat bran, 15.7% CP	70	CP, g/kg	189.9
Barley	100	CF, g/kg	34.5
Soybean meal, 42.0% CP	250	Ash, g/kg	59
Fish meal, 64% CP	25	Fat, g/kg	70.8
Soybean oil	45	Ca, g/kg	9.5
Dicalcium phosphate	5	Total P, g/kg	8.4
Limestone	12	Available P, g/kg	5.7
Salt	3	Digestible Lys, g/kg	11.3
Sodium bicarbonate	2	Digestible Met + Cys, g/kg	5.8
Sodium sulfate	4	Digestible Thr, g/kg	8.5
Vitamin and mineral premix^a^	8	Digestible Trp, g/kg	2.2
Choline choride (50%)	2		
Vitamin C (95%)	0.2		
Vitamin E (50%)	0.2		
L-Lys**·**HCL	1.5		
L-Thr	1.5		
Total	1000		

^a^Provided the following per kilogram of diet: 24000 IU of vitamin A, 3000 IU of vitaminD3, 60 mg of vitamin E, 5 mg of vitamin K, 5 mg of vitamin B1, 12.5 mg of vitamin B2, 24 mg of pantothenic acid, 50 mg of niacin, 5 mg of vitamin B6, 0.037 mg of vitamin B12, 2.2 mg of folacin, 0.1 mg of biotin, 8 mg of Cu, 60 mg of Fe, 35 mg of Mn, 65 mg of Zn, 0.35 mg of I, 0.3 mg of Se

### Milk collection

Maningat et al. [41] demonstrated that the RNA obtained from the milk fat globule was suitable to determine the gene expression profile of human mammary epithelial cells during lactation. The same method was also used in the rat [42], dairy cow [43], human [11, 44–46], and buffalo [47]. Consequently, milk fat globule RNA was used to determine gene expression in this research in lieu of mammary gland biopsies. Colostrum samples were collected immediately at 0, 2, 6, 12, 24 and 48 h after birth of the first piglet. Milk samples were collected on day 3, 4, 7, 14, and 21 of lactation following the intramuscular injection of 20 IU oxytocin (each ampule contained 20 IU oxytocin) at the neck muscle of sows to facilitate milk letdown. Each 20 mL sample was collected from 3 functional mammary glands (anterior, middle, and posterior) on each side of the udder by hand milking. Samples were immediately stored in liquid nitrogen for subsequent analysis. The milk samples were centrifuged at 3500 rpm for 15 min at 4 °C as described by Mohammad et al. [11]. The supernatant fat layer was transferred into new tubes, and 2 mL TRIzol (Invitrogen, Carlsbad, CA, USA) was added into the tube. The fat and TRIzol mix was homogenized, and stored at − 80 °C. The infranatant was also stored at − 80 °C for subsequent analysis.

### Milk yield

The estimation of the milk yield on days 2, 7, 14, and 21 of lactation was based on the equation described by Hansen et al. [48]. Litter size and litter gain were recorded for days 2, 7, 14, and 21 in lactation and used as inputs to predict the milk yield.

### Milk lactose and hormone analyses

Milk lactose concentration was determined by Lactose/D- galactose (Rapid) Assay kit (Megazyme international Ireland Ltd., Wicklow, Ireland). Prolactin, progesterone, insulin (1μIU/mL = 1 ng/mL× 21.2), and IGF-1 content in milk infranatant were determined by radioimmunoassay (RIA) method using commercial kits (human, converted to pig estimates) (Tianjin Jiuding Medical and Biological Engineering Co., Ltd., Tianjin, China) as described by Foisnet et al. [49].

### RNA extraction and qPCR

Total RNA was isolated from the mix of milk fat and TRIzol reagent (Invitrogen, Carlsbad, CA, USA) according to the manufacturer’s instructions. Total RNA concentration and purity were measured using Nano Drop spectrophotometer (Nano Drop Technologies, Wilmington, DE, USA). The rate of A260/280 was from 1.95 to 2.10; A260/230 was from 1.78 to 2.11. The genomic DNA elimination and the cDNA synthesis was performed according to the instructions of a Prime Script RT reagent kit (Takara, Dalian, Liaoning, China). The quantitative real-time PCR was executed on the ABI Prism 7500 Sequence Detection System (Applied Biosystems, Carlsbad, CA, USA) in a volume of 20 μL. SYBR Green Real-Time PCR Master (Toyobo, Osaka, Japan) was the reaction mix. GAPDH, TBP, MRPL39, β-actin, and SDHA was selected by geNorm 3.5 (http://medgen.ugent.be/~jvdesomp/genorm/) as the reference genes for the qPCR. Gene specific forward and reverse primers were designed using Primer Premier 5 (PREMIER Biosoft Int., Palo Alto, CA, USA). All the primers of the target gene and reference gene for qPCR were shown in Table 2.

**Table 2:** Primers used for RT-PCR

Gene name	Gene accession	Primer sequence (5′-3′)	Size (bp)
GLUT1	EU012358	F: GATGAAGGAGGAGTGCCG	106
		R: CAGCACCACGGCGATGAGGAT	
SGLT1	NM001164021	CATCATCGTCCTGGTCGTC	138
		GGGGCTTCTTGAATGTCCT	
HK1	XM003359224	CGAGATAACAAGAGCACACCC	138
		GAGGAAACGCACATCACAGTC	
HK2	DQ432056	GTTCCTGGCTCTGGATCTTGG	115
		GGGATGGCGTAGATCTGGTTC	
LALBA	NM214360	GTGGTGGGGATTCTCTTTCC	179
		TCTGTGCTGCCATTGTCATG	
B4GALT1	XM003130680	GAGTTTAACATGGCCGTGGAC	185
		TGACGCTGTAGGATTGGGTG	
PGM1	NM001246318	TGGTCGCTTGGTTATTGG	123
		TTGGGTCCTCCTGGGTTGT	
PGM2	XM003128910	CAACAGTGACGCACCCGA	198
		GCCACCAGCCCAAGAGG	
UGP2	NM213980	TACCACGGCACCATCACA	238
		GGTTCCCAAACCACCATT	
GALT	XM005668043	TGGTTGGCTACGAAATGC	126
		TGGCTGCTGTCTCCTTGT	
GALE	XM003356202	GACTATGACACGGAGGATGG	108
		CGTGCCCAGGTTGTAGAT	
SLC35A2	XM013986120	GCTGTGGTCATGGCTGAAGT	196
		TGGAGATGGCAACATACTGGA	
AKT1	NM001159776	CCTGAAGAAGGAGGTCATCG	123
		TCGTGGGTCTGGAAGGAGTA	
PRLR	NM001001868	TTTCTGCTGTCGTCTGTTTGA	142
		TCTTCGGACTTGCCTTTCTC	
STAT5a	NM214290	CATCACCATTGCCTGGAAG	141
		CGGTCGGGAAACACATAGAT	
STAT5b	NM214168	TGTGAGAAGTTGGCGGAGAT	169
		CGATGATGAATGTGCTGGTC	
ACACA	NM001114269	ACATCCCCACGCTAAACA	186
		AGCCCATCACTTCATCAAAG	
FASN	NM001099930	GCTTGTCCTGGGAAGAGTGTA	115
		AGGAACTCGGACATAGCGG	
PGLS	XM003123494	GCTGGACTCTGGGCTTCTG	138
		GCAGCTCAGGGTTAATGGTG	
β-ACTIN	XM003124280	GGATGCAGAAGGAGATCACG	105
		ATCTGCTGGAAGGTGGACAG	
GAPDH	NM001206359	AAGGTCGGAGTGAACGGATT	248
		CATTTGATGTTGGCGGGAT	
MRPL39	AY610067	CAAAAGAGAACCTACATTCCTTCACA	100
		TCTAATGCCACTTTTGCTTCAACT	
SDHA	DQ402993	ACTCGCTCCTGGACCTCGT	152
		GGTTCCGTTCGCAAATCTC	
TBP	DQ178129	GATGGACGTTCGGTTTAGG	124
		AGCAGCACAGTACGAGCAA	

All the expression levels were normalized by the arithmetic mean of the selected control gene (GAPDH, TBP, MRPL39, β-actin, or SDHA). The mRNA expression was calculated using the 2^-ΔΔCt^ method [50].

### Western blot analysis

Primary antibodies (dilution, cat. no. follow in parentheses) for GLUT1 (1:500, ab150299), HK1 (1:1000, ab209661), UGP2 (1:1000, ab154817), PGM1 (1 μg/mL, ab94601), and B4GALT1 (1:1000, ab211207) were from the Abcam Company Ltd. (Cambridge, MA, USA). The primary antibody for SLC35A2 (1:200, sc-82,031) was from Santa Cruz Biotechnology (Delaware Ave Santa Cruz, CA, USA). The secondary antibodies (1:6000; except PGM1, 1:50000) were from Cell Signaling (Danvers, MA, USA). The protein concentrations were measured using Pierce BCA protein assay kits (Thermo Fisher Scientific, Rockford, IL, USA). The Western blot analysis procedure was conducted according to Lv et al. [39]. The milk samples were electrophoresed on 10% polyacrylamide gel and then transferred onto nitrocellulose membranes (Millipore, Bedford, MA, USA). The membranes were blocked for 3 h with Tris-buffered saline solution containing 0.1% Tween 20 (TBST) containing 5% fat free milk and incubated with primary antibodies at 4 °C overnight (over 12 h). Subsequently, the membranes were washed by TBST and incubated with secondary antibody for 1 h at room temperature. Western blots were then generated with an enhanced electrochemiluminescence reagents (ECL) (Beyotime, Shanghai, China). The bands were quantified by Image Processing Software (Image Pro Plus 6.0) (Rockville, MD, USA).

### Statistical analysis

Statistical evaluation of milk yield, lactose and hormones concentration in milk, transcription, and western blot was performed using the ANOVA and LSD procedure of SPSS 19.0 software (SPSS Inc., Chicago, IL). Data were analyzed as a randomized complete block with a linear model that included sow (random), time (fixed, repeated), sow x time (random error), and among samples within sow (random subsample variance). Differences at *P* < 0.05 were considered statistically significant. Data were expressed as means ± SEM.

## Results

### Milk yield and lactose concentration and yield

**Fig. 1:**
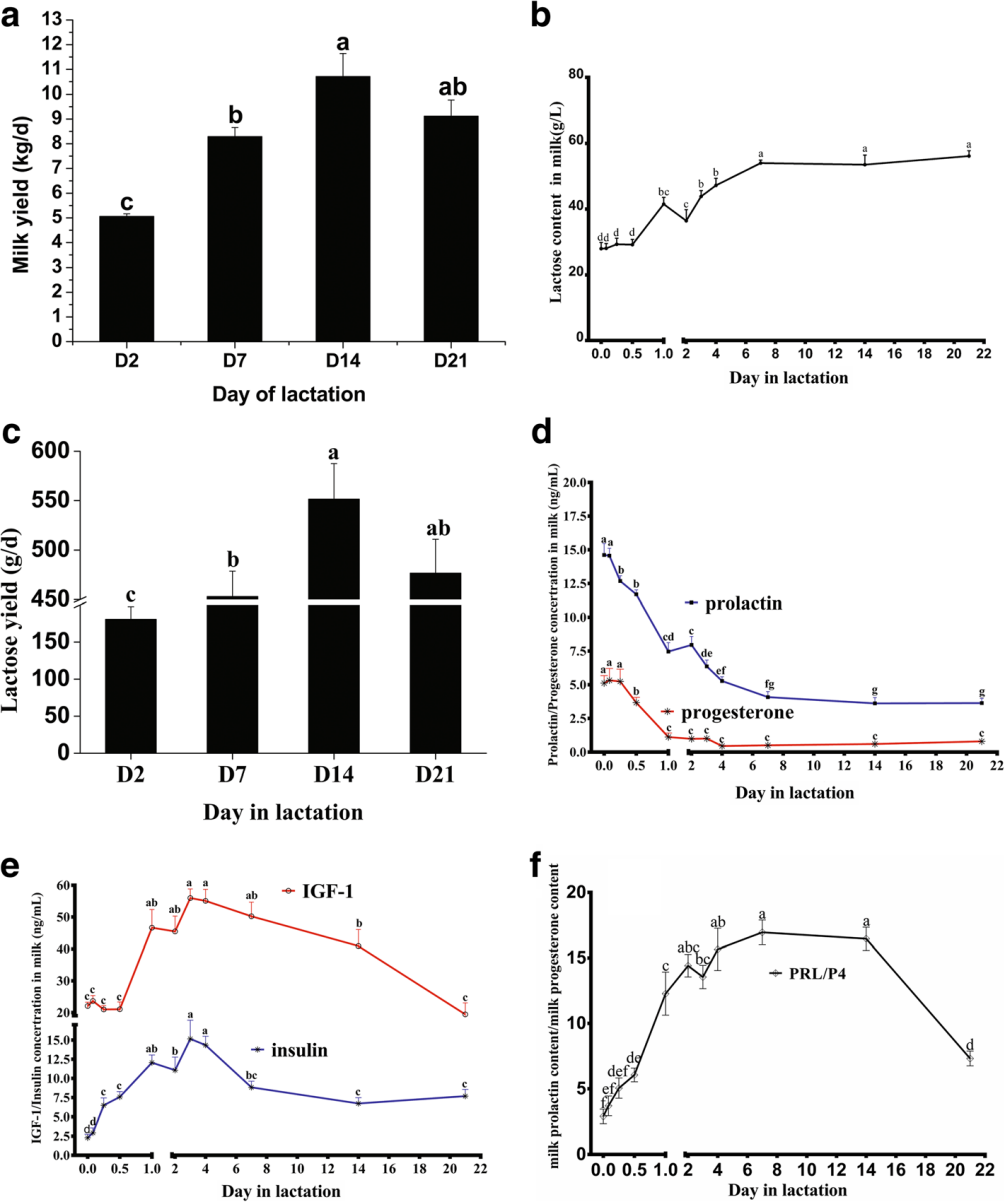
The milk yield (**a**), lactose yield (**b**), milk lactose concentration(**c**), milk prolactin and progesterone concentration (**d**), milk insulin and IFG-1 concentration (**e**), and ratio of milk prolactin content and milk progesterone content (**f**) of lactating sows. Values are means ± SEM (*n* = 8); Labeled means without a common letter differ, *P* < 0.05

Milk yield increased linearly from D2 to D14 (5.06 vs 10.71 kg/d, *P* < 0.05), and decreased at D21 of lactation (compared to D14, *P* > 0.05) (Fig. 1a). There was no difference in the lactose concentration from 0 h to 12 h after the first piglet was born (*P* > 0.05, Fig. 1b). Lactose concentration increased significantly from 12 h to D7 (29.12 vs 54.02 g/L) (*P* < 0.01), reaching a relative plateau, with the maximum concentration of 56.17 g/L at D21 of lactation. Similar to milk yield, lactose yield of sows increased 2-fold from D2 to D14 (180.70 vs 551.61 g/d, *P* < 0.05), and then decreased at D21 (476.66 g/d) in lactation (Fig. 1c).

### Hormone concentration in colostrum or milk

To explore the lactose synthesis mechanism, concentrations of four hormones related to lactose synthesis were measured in milk (prolactin, progesterone, insulin, and IGF-1). The milk prolactin and progesterone concentrations declined progressively from 0 h (prolactin: 14.56 ng/mL; progesterone: 5.24 ng/mL) and plateaued at D7 (prolactin: 4.09 ng/mL) or D1 (progesterone: 1.12 ng/mL), and then remained relatively constant throughout the remainder of lactation (Fig. 1d). However, milk insulin and IGF-1 concentration increased significantly in early lactation to D3, and then decreased thereafter. Insulin increased from 2.28 ng/mL at 0 h to a maximum of 15.17 ng/mL on D3 (*P* < 0.05) with a decline thereafter to D7 (*P* < 0.05), when it plateaued (Fig. 1e). The milk IGF-1 concentration increased 2-fold from 12 h (21.06 ng/mL) to D3 (55.97 ng/mL), and then decreased to 19.24 ng/mL at D21 (*P* < 0.05, Fig. 1e).

### Expression of genes or proteins related to lactose synthesis in milk throughout the whole lactation

**Fig. 2:**
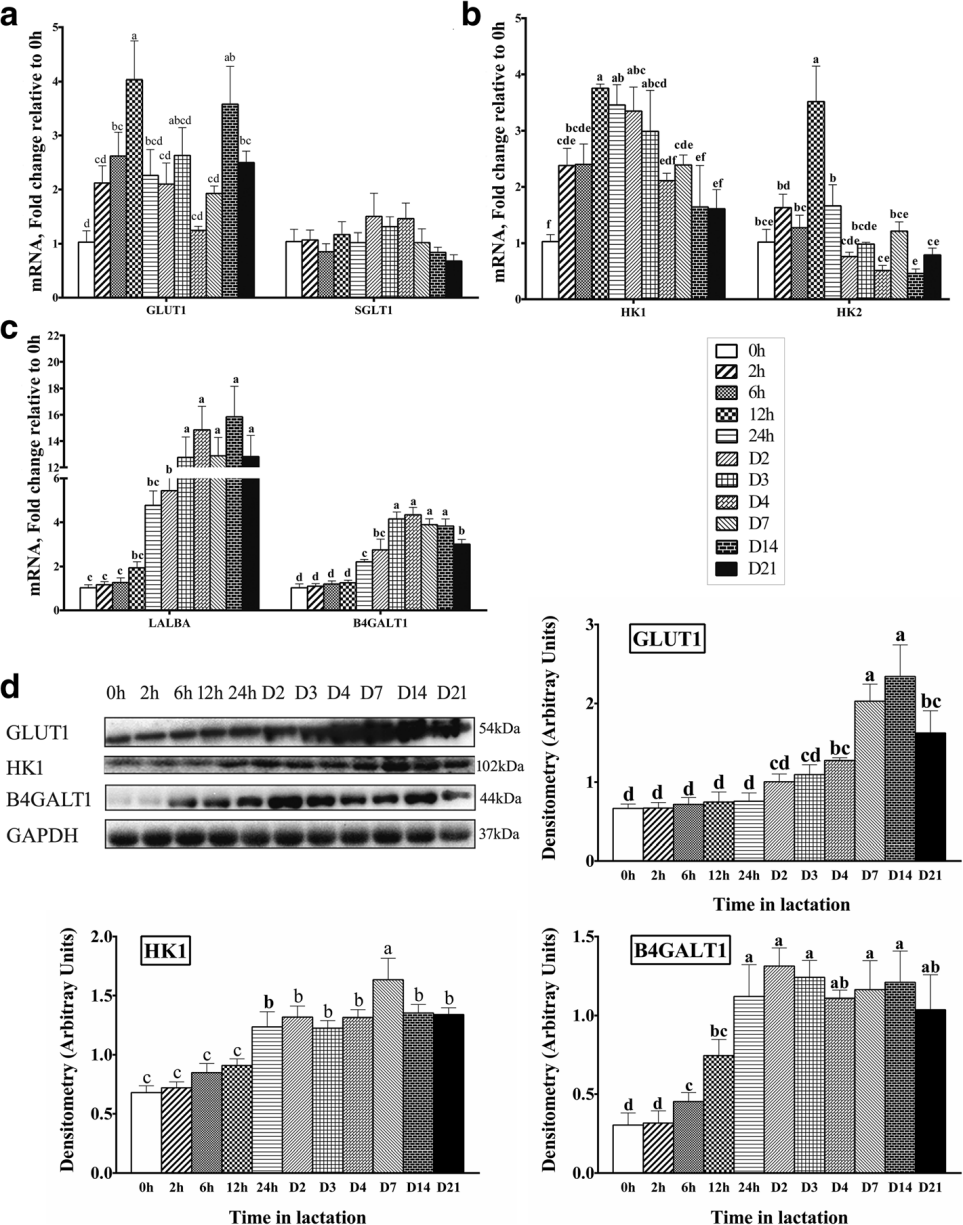
The expression of genes or proteins for GLUT1, SGLT1, HK1, HK2, LALBA, and B4GALT1 in milk throughout lactation. **a** The mRNA expression of genes for GULT1 and SGLT1 (*n* = 8). **b** The mRNA expression of genes for HK1 and HK2 (n = 8). **c** The mRNA expression of genes for LALBA and B4GALT1 (n = 8). **d** Western blot analysis for GLUT1, HK1, and B4GALT1 (*n* = 4). Values are means ± SEM; Labeled means without a common letter differ, *P* < 0.05

The mRNA expression of GLUT1 increased from 0 h to 12 h (*P* < 0.05), and then decreased and was relatively stable to D14 when it increased to levels comparable to 12 h (*P* < 0.05, Fig. 2a). Compared with 0 h, the mRNA expression of GLUT1 increased 1.62-fold at 6 h, 3-fold at 12 h, 2.6-fold at D14 and 1.5-fold at D21 respectively (*P* < 0.05). However, there was no change in the mRNA expression of SGLT1 throughout lactation (*P* > 0.05, Fig. 2a). Western blot analysis showed that GLUT1 markedly increased from 0 h to D14 (*P* < 0.05), but then declined at D21 (Fig. 2d). The GLUT1 protein expression increased 0.92-fold at D4, 2-fold at D7, 2.5-fold at D14, and 1.45-fold at D21 (*P* < 0.05) from 0 h of lactation.

The transcription abundance of HK1 increased from 0 h to 12 h (*P* < 0.05) to the maximum value, and then gradually decreased during the remainder of lactation (*P* < 0.05, Fig. 2b). The mRNA expression of HK1 was upregulated 1.38-fold at 2 h, 1.4-fold at 6 h, 2.76-fold at 12 h, 2.46-fold at 24 h, 2.35-fold at D2, 1.99-fold at D3, and 1.39-fold at D7 (*P* < 0.05) compared with 0 h. Similarly, the HK1 protein expression increased 1.4-fold (*P* < 0.05) from 0 h to D7 (Fig. 2d), and then slightly declined at D14 and D21 from D7 (*P* < 0.05) of lactation. However, the mRNA expression of HK2 was increased from 0 h only at 12 h (2.52-fold, *P* < 0.05), there was little difference between 0 h and other times of lactation.

The changes in genes of LALBA and B4GALT1 involved in lactose synthetase were similar to the milk lactose concentration profile (Fig. 2c). The mRNA expression of LALBA and B4GALT1 was upregulated from 0 h to D3 (*P* < 0.05), and reached a relative plateau from D3 to D21 or D14. The transcript abundance of LALBA was significantly upregulated by 4.44-fold at D2, 11.76-fold at D3, 13.85-fold at D4, 11.88-fold at D7, the peak value (14.84-fold) at D14, and 11.82-fold at D21 respectively (*P* < 0.05). Compared with 0 h, the mRNA expression for B4GALT1 was augmented 1.21-fold at 24 h, 1.76-fold at D2, 3.15-fold at D3, 3.34-fold at D4, 2.89-fold at D7, 2.83-fold at D14, and 2.02-fold at D21 respectively (*P* < 0.05). Furthermore, the protein expression of B4GALT1 also increased 3.3-fold by D2 from 0 h (Fig. 2d), and attained to a relative plateau from D1 to 21 of lactation.

### Expression of genes or proteins related to glucose-galactose interconversion and UDP-galactose synthesis and transport in milk throughout the whole lactation

**Fig. 3:**
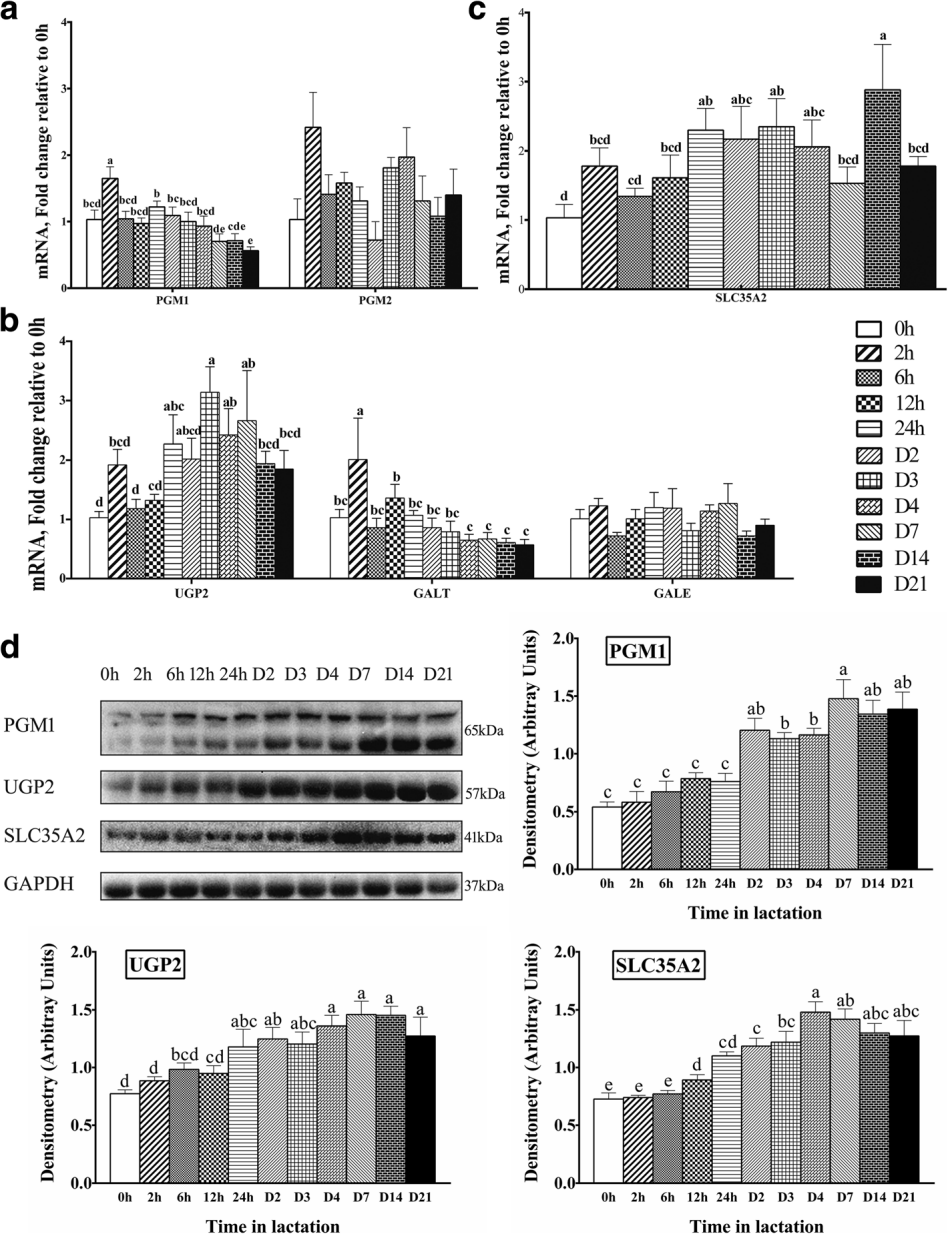
The expression of gene or proteins for glucose-galactose interconversion and UDP-galactose synthesis and transport in milk throughout lactation. **a** The expression of genes for PGM1, and 2 (*n* = 8). **b** The expression of genes for UGP2, GALT and GALE (*n* = 8). **c** The expression of genes for SLC35A2 (*n* = 8). **d** Western blot analysis for PGM1, UGP2, and SLC35A2 (n = 4). Values are means ± SEM; Labeled means without a common letter differ, *P* < 0.05

The mRNA expression of genes related to glucose-galactose interconversion (PGM1, PGM2) and UDP-galactose synthesis (UGP2, GALT, GALE) and transportation (SLC35A2) in milk throughout the whole lactation is shown in Fig. 3. The mRNA expression of PGM1 was significantly upregulated (1.65-fold, *P* < 0.05) at 2 h, but then decreased gradually, with a decrease of nearly 50% at D21 compared to 0 h. There was no significant change in the mRNA expression abundance of PGM2 in the whole lactation (Fig. 3a). At variance with the mRNA expression, the protein expression of PGM1 increased 1.73-fold by D7 compared to 0 h (*P* < 0.05), and then reached a relative plateau from D7 to D21.

The mRNA abundance of UGP2 increased slowly from 0 h, reached the maximum value at D3 (3-fold, *P* < 0.05), and then decreased from D3 to D21 (Fig. 3b). The mRNA expression of UGP2 increased 1.27-fold at D1, 2.14-fold at D3, 1.42-fold at D4, and 1.66-fold at D7 (*P* < 0.05). The protein expression of UGP2 increased slowly to D7 (89%, *P* < 0.05) compared to 0 h (Fig. 3d). The mRNA expression of GALT increased at 2 h (1-fold, *P* < 0.05), and then gradually decreased. There was no significant change in the mRNA expression abundance of GALE during lactation (*P* > 0.05, Fig. 3b).

The mRNA expression of SLC35A2 increased 1.88-fold by D14 from 0 h (*P* < 0.05), and decreased at D21 from D14 (*P* < 0.05, Fig. 3c). Similarly, the protein expression of SLC35A2 increased 1-fold by D4 (*P* < 0.05), reaching a relative plateau thereafter (Fig. 3d).

### Expressions of genes related to regulation of transcription in milk throughout the whole lactation

**Fig. 4:**
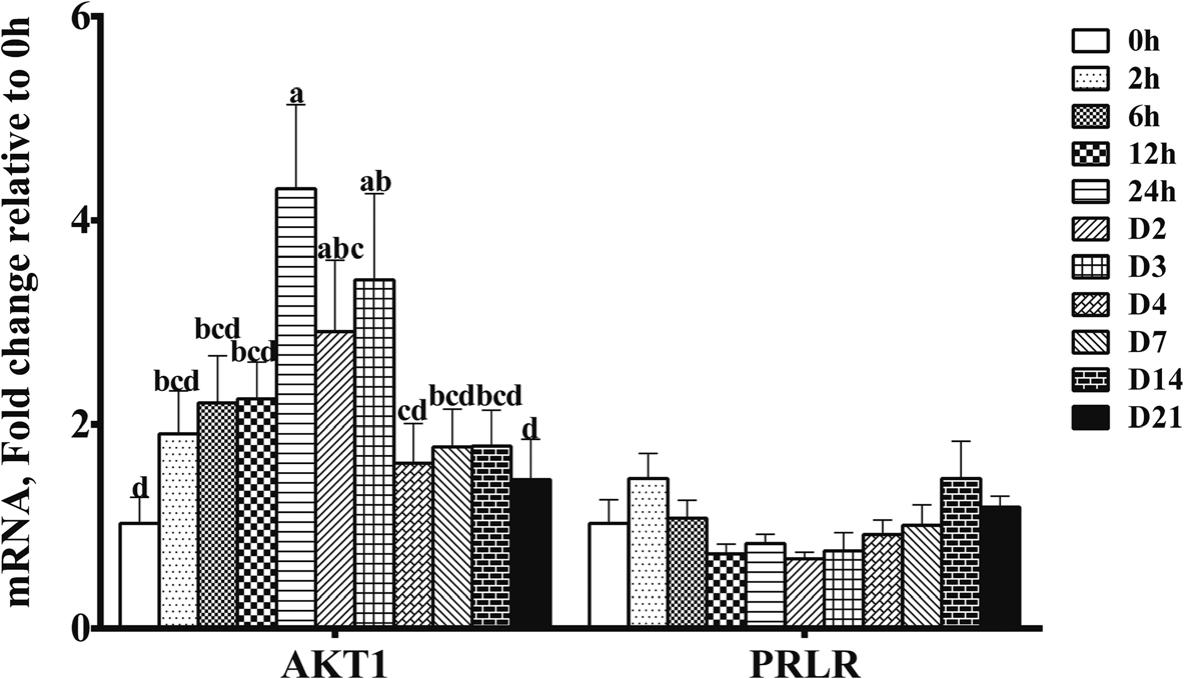
Expression of transcription factors for AKT1 and PRLR at mRNA level in milk throughout lactation. Values are means ± SEM (*n* = 8); Labeled means without a common letter differ, *P* < 0.05

The mRNA expression of AKT1 increased at D1 (*P* < 0.05) (Fig. 4), then decreased and reached a relative plateau at D4. The mRNA abundance for AKT1 increased 3.31-fold at D1, 1.91-fold at D2, and 2.42-fold at D3 (*P* < 0.05). However, there was no significant change for PRLR mRNA expression throughout lactation.

## Discussion

As a key osmotic agent, lactose synthesis is an important factor in milk production [10] and has an indirect influence on milk protein and fat yield [13, 14]. Reports have shown that, from day 1 to day 14 postpartum in sows, milk production and concentration of milk lactose increased approximately 1-fold [7, 48, 51]. Consistent with these previous reports, we found milk yield and milk lactose concentration increased 1-fold and lactose yield increased 3-fold in the present study. Incremental lactose yield and up regulated lactose synthesis was likely attributable to the upregulation of genes involved in the lactose synthesis pathway, including glucose uptake, glucose-galactose interconversion, glucose and UDP-galactose transportation, lactose synthesis, and corresponding transcription regulation.

As the primary substrate for lactose synthesis, glucose contributes approximately 80% of the carbons in lactose in humans [25] and 85% in cows [26, 27]. However, mammary glands are unable to synthesize glucose from other precursors due to the shortage of glucose-6-phosphatase [52]. Any glucose existing in lactating mammary glands has been shown to be fully transported from blood [16]. Two glucose transporters, glucose transporter 1 (GLUT1) and Na^+^ − coupled glucose transporter 1 (SGLT1), were found to participate in this transport [18, 33, 53]. During lactation, expression of GLUT1 was reported to be significantly increased in humans [11], bovines [15, 35, 54], rats [24, 30], and goats [31]. Similarly, in sows, a significant increase of GLUT1 mRNA and protein expression was observed in the mammary gland from late pregnancy to peak lactation [32, 33]. In this study, we evaluated the temporal gene expression changes of GLUT1 and SGLT1 during the whole lactation. There was no significant change in the abundance of SGLT1 during this period. However, we found that the protein expression of GLUT1 gradually increased from 0 h to D14 (1.8-fold) after parturition and was relatively stable until the end of lactation. Based on reports from the literature and results of our research it is evident that glucose transporters (such as GLUT1) are involved in one of crucial steps of lactose synthesis during lactation.

Once glucose enters cells, it is converted into glucose-6-phosphate through hexokinase (HK). Xiao and Cant [34] reported that phosphorylation by hexokinase exerted 80% of the control of glucose metabolism to lactose in bovine mammary epithelial cells, and hexokinase was likely the limiting step in the lactose synthesis pathway. There are four isozymes of hexokinase, but, only HK1 and HK2 have been reported to be expressed in the mammary gland [19]. Chen et al. [33] observed that the mRNA expressions of HK1 and HK2 were upregulated in the mammary gland at D1 of lactation compared with pregnancy. In this study, we evaluated the temporal protein expression change of HKs in the whole lactation. We found that the protein expression of HK1 was increased gradually during the lactation. The results in the present study indicated that HKs are likely the limiting step in early lactation.

**Fig. 5:**
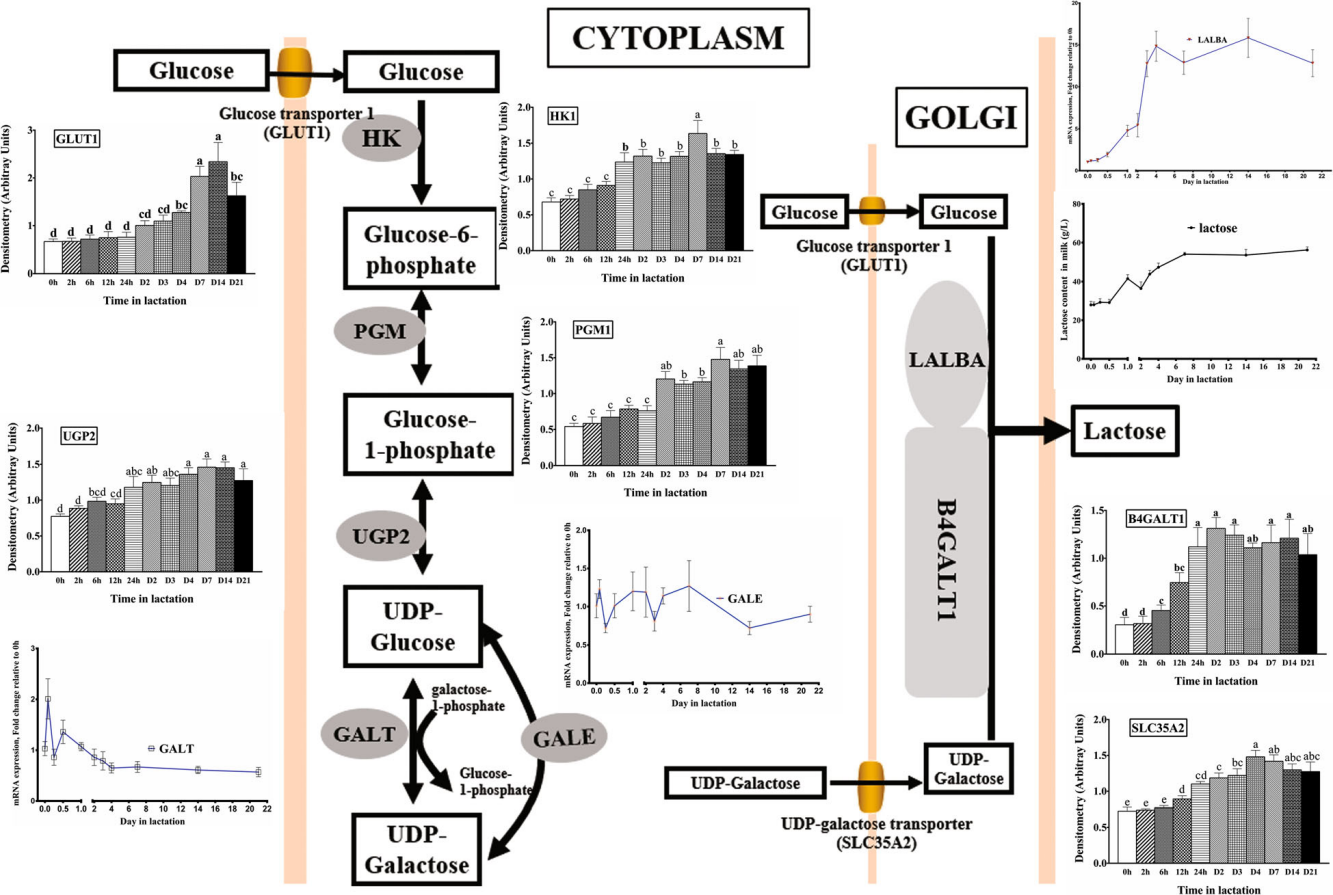
The gene or protein expression of GLUT1, HK1, PGM1, UGP2, GALT, GALE, SLC35A2, LALBA, and B4GALT1 on the lactose synthesis pathway throughout lactation. The mRNA or protein expressions of GLUT1, HK1, PGM1, UGP2, SLC35A2, LALBA, and B4GALT1 were upregulated (*P* < 0.05). The mRNA expression of GALE did not change, and the mRNA expression of GALT was downregulated

Once glucose is phosphorylated to glucose-6-phosphate, it is subsequently converted into UDP-galactose in the cytoplasm by UGP2, PGMs, GALT, and GALE, and enters the Golgi bodies via SLC35A2 [10, 11]. In this research, we observed that the protein abundance of PGM1 increased progressively postpartum and reached a maximum value at D7 of lactation. This finding is in agreement with results in humans [11] and bovines [55]. We also observed that both mRNA and protein expression of UGP2 and SLC35A2 increased during lactation in the present study. Similar results were observed in sows [32], humans [11], and bovines [56] in previous reports. However, the transcript abundance of GALT increased at 2 h after the birth of the first piglet and then decreased linearly during the subsequent lactation in our study, which differed from the results in humans [11]. The uncoordinated regulation of genes encoding for the UDP-galactose synthesis and transportation pathway in the present study (Fig. 5) indicated that they were likely not the limiting step in the lactose synthesis pathway in lactating sows.

Lactose is synthesized by lactose synthetase from glucose and UDP-galactose when they are transported into Golgi bodies by glucose transporter GLUT1 and UDP-galactose transporter SLC35A2. Lactose synthetase is composed of two subunits (LALBA and B4GALT1), both of which are required for activity [57, 58]. β 1,4-Galactosyltransferase-I is one of seven β 1,4-Galactosyltransferases and adds galactose to oligosaccharides [59]. α-lactalbumin (LALBA) promotes the glucose binding to B4GALT1and increases the B4GALT1 activity by approximately 30-fold [60–63]. Bleck et al. [64] reported that LALBA concentration positively correlated with the milk concentrations of protein, fat, and lactose. Many previous reports also found that the mRNA or protein expression of LALBA increased from pregnancy to lactation in rats [24], humans [46], bovines [37, 56], goats [36, 65], and sows [33]. Consistent with former studies, the transcript abundance of LALBA was augmented significantly during lactation and increased 14-fold at D14 in this study. Similarly, the mRNA and protein expressions of B4GALT1 were also strongly upregulated in lactation. This finding was consistent with reports in rats [24], bovines [37, 64], and sows [33]. The transcript abundances or protein expressions of LALBA and B4GALT1 increased with lactose yield and milk concentration of lactose in this study (Fig. 5), indicating that lactose synthetase might play an important role and be the rate-limiting step in lactose synthesis in lactating sows.

Lactogenic hormones such as prolactin, insulin, and growth hormone are necessary to induce and maintain successful lactation [66]. Prolactin deficiency decreased milk production and lactose yield in rats [67] and cows [68], which perhaps was related to the direct effect of prolactin on the opening of tight junctions between mammary epithelial cells [67], inhibiting epithelial cell loss and maintaining cellular differentiation [67, 68]. In humans, prolactin has been reported to increase lactose synthesis at initiation of lactation via the PRLR-JAK2-STAT5 signaling pathway upregulating the mRNA expression of UGP2 and SLC35A2 [11]. Prolactin also has been reported to maintain GLUT1 level [69] and upregulate glucose intake [70] for increased lactose yield via the PI3K-AKT1 signaling pathway [70]. The activation of AKT1 was necessary and sufficient to STAT5 activation [71]. Prolactin has been reported to enhance the activity of B4GALT1and LALBA and increase the tissue accumulation of the mRNA of both [72]. As is known, insulin can strongly upregulate the activation of STAT5 [73], which indirectly upregulates the gene expression of LALBA with the promoter region of LALBA containing a STAT5 binding site [10]. Prolactin, combined with insulin, hydrocortisone, and estradiol, increased the mRNA expression of LALBA several hundred-fold in bovine mammary epithelial cell [74]. It has been shown to be an important hormone regulating the lactogenic and galactopoietic processes [75]. However, in the present study, the milk concentration of prolactin and progesterone decreased gradually, which indicated the role of prolactin on lactose synthesis may less important in sows. In addition, as the downstream gene in the prolactin pathway, the transcript abundance of PRLR did not change significantly in our study. Only the mRNA expression of AKT1 increased in early lactation. The observations in this study are consistent with a previous report in our lab [33]. The evidence of the relationship between increased lactose synthesis and milk prolactin concentration or the expression of AKT1 and PRLR suggested that prolactin or the regulating factors played minor roles in lactose synthesis in lactating sows. Loisel et al. [76] reported that the relative prolactin-to-progesterone concentrations influenced the colostrum yield in sows and indicated the inducing role of prolactin on the onset of lactose synthesis. In the present study, the ratio of prolactin-to-progesterone concentration increased linearly in the early lactation, which indicated that prolactin and decreasing progesterone might be related to the onset of lactation.

Reports indicate that both prolactin and growth hormone can regulate milk composition synthesis [67], and growth hormone is likely regulated through the production of IGF-1 [77, 78]. IGF-1 can induce ductal growth and cell proliferation in the mammary gland [78–80] and play an important role in mammary morphogenesis [81]. In the current study, the milk IGF-1 concentration increased in early lactation. This finding indicates that the growth of mammary tissue occurs during early lactation, which is consistent with previous research [82]. It is reasonable to hypothesize that IGF-1 induces mammary gland growth to which increases lactose synthesis in lactating sows.

## Conclusion

In summary, milk and lactose yield gradually increased with the progression of lactation in sows. Lactose synthesis was not significantly influenced by lactogenic hormones (e.g., prolactin, insulin, and IGF-1). The transcript abundances or protein expressions of GLUT1, LALBA, and B4GALT1 increased significantly along with increased lactose concentration and yield during lactation, indicating that they might be important in critical steps in lactose synthesis in lactating sows.

## Abbreviation

AKT: Protein kinase B
B4GALT1: β 1,4-Galactosyltransferase-1
GALE: UDP-galactose-4-epimerase
GALT: Galactose-1-phosphate uridylyltransferase
GLUT1: Facilitate glucose transporter 1
HK: Hexokinase
IGF-1: Insulin like growth factor-1
JAK: Janus kinase
LALBA: α-lactalbumin
PGM1: Phosphoglucomutase 1
PI3K: Phosphatidylinositol 3 kinase
PRLR: Prolactin receptor
qPCR: Quantitative PCR
SGLT1: Sodium-dependent glucose transporter 1
SLC35A2: UDP-galactose transporter
STAT: Signal transducers and activators of transcription
UDP: Uridine diphosphate
UGP2: UDP-glucose pyrophosphorylase 2
